# Primary Omental Infarction Mimicking Acute Appendicitis: A Case Report

**DOI:** 10.7759/cureus.107843

**Published:** 2026-04-27

**Authors:** Amani Alabdouli, Aysha Al Harthi, Hind Aldhaheri

**Affiliations:** 1 Emergency Medicine, Tawam Hospital, Al Ain, ARE

**Keywords:** abdominal pain, acute abdomen, conservative management, ct abdomen, omental infarction

## Abstract

Omental infarction (OI) is a rare cause of acute abdominal pain that often mimics common surgical emergencies such as appendicitis. Due to its nonspecific presentation, the diagnosis is frequently delayed or missed. We report the case of a 43-year-old man who presented with right lower quadrant abdominal pain and was initially suspected to have appendicitis. Contrast-enhanced computed tomography (CT) revealed findings consistent with primary OI. The patient was managed conservatively with analgesics and anti-inflammatory medications, resulting in complete symptom resolution within one week. This case highlights the importance of considering OI in patients presenting with localized abdominal pain and normal laboratory findings, as early radiologic diagnosis allows for conservative management and the avoidance of unnecessary surgical intervention.

## Introduction

Omental infarction (OI) is an uncommon cause of acute abdominal pain, accounting for <1% of acute abdomen presentations in the adult population [1]. It results from vascular compromise of the greater omentum, leading to ischemia and inflammation. Clinically, OI often mimics more common surgical conditions such as appendicitis or cholecystitis, making diagnosis challenging [2]. With the increasing availability of computed tomography (CT), preoperative recognition has improved, allowing conservative management in most cases. We present a case of primary OI diagnosed on CT in a patient with right lower quadrant pain, emphasizing the importance of imaging in avoiding unnecessary surgical intervention.

## Case presentation

A 43-year-old male patient presented to the emergency department with a three-day history of right lower quadrant abdominal pain. The pain was acute in onset, sharp, localized, nonradiating, constant, and progressively worsening, aggravated by movement. He denied fever, nausea, vomiting, or diarrhea.

He had no significant past medical or surgical history and was not taking regular medications. On examination, he appeared uncomfortable but was hemodynamically stable (temperature 36.8°C, blood pressure 120/80 mmHg, heart rate 82 bpm, respiratory rate 18 breaths/min). Abdominal examination revealed localized tenderness in the right lower quadrant without guarding or rebound tenderness.

Laboratory investigations in Table 1 were within normal limits except for an elevated C-reactive protein (25.7 mg/L). Urinalysis was unremarkable.

**Table 1:** Laboratory investigations AST, aspartate aminotransferase; ALT, alanine aminotransferase; ALP, alkaline phosphatase

Test	Result	Reference range
WBC	10.1×10⁹/L	4.0-11.0
Hemoglobin	14.2 g/dL	13-17
RBC	4.92×10¹²/L	4.5-5.9
Hematocrit	0.42	0.40-0.50
Platelets	314×10⁹/L	150-400
Neutrophils	59.30%	40-75
Lymphocytes	31.20%	20-45
Monocytes	8.00%	2-10
Eosinophils	1.10%	0-6
CRP	25.7 mg/L	<5
Sodium	140 mmol/L	135-145
Potassium	4.0 mmol/L	3.5-5.0
Chloride	104 mmol/L	98-107
CO₂	25 mmol/L	22-29
Creatinine	65 µmol/L	60-110
Urea	2.55 mmol/L	2.5-7.1
AST	15 U/L	<40
ALT	<5 U/L	<41
ALP	100 U/L	40-130
Bilirubin total	8.5 µmol/L	<21
Urinalysis	Normal	-
Urine nitrite	Negative	-
Urine leukocytes	Negative	-
Urine protein	Negative	-
Urine glucose	Negative	-

**Figure 1:**
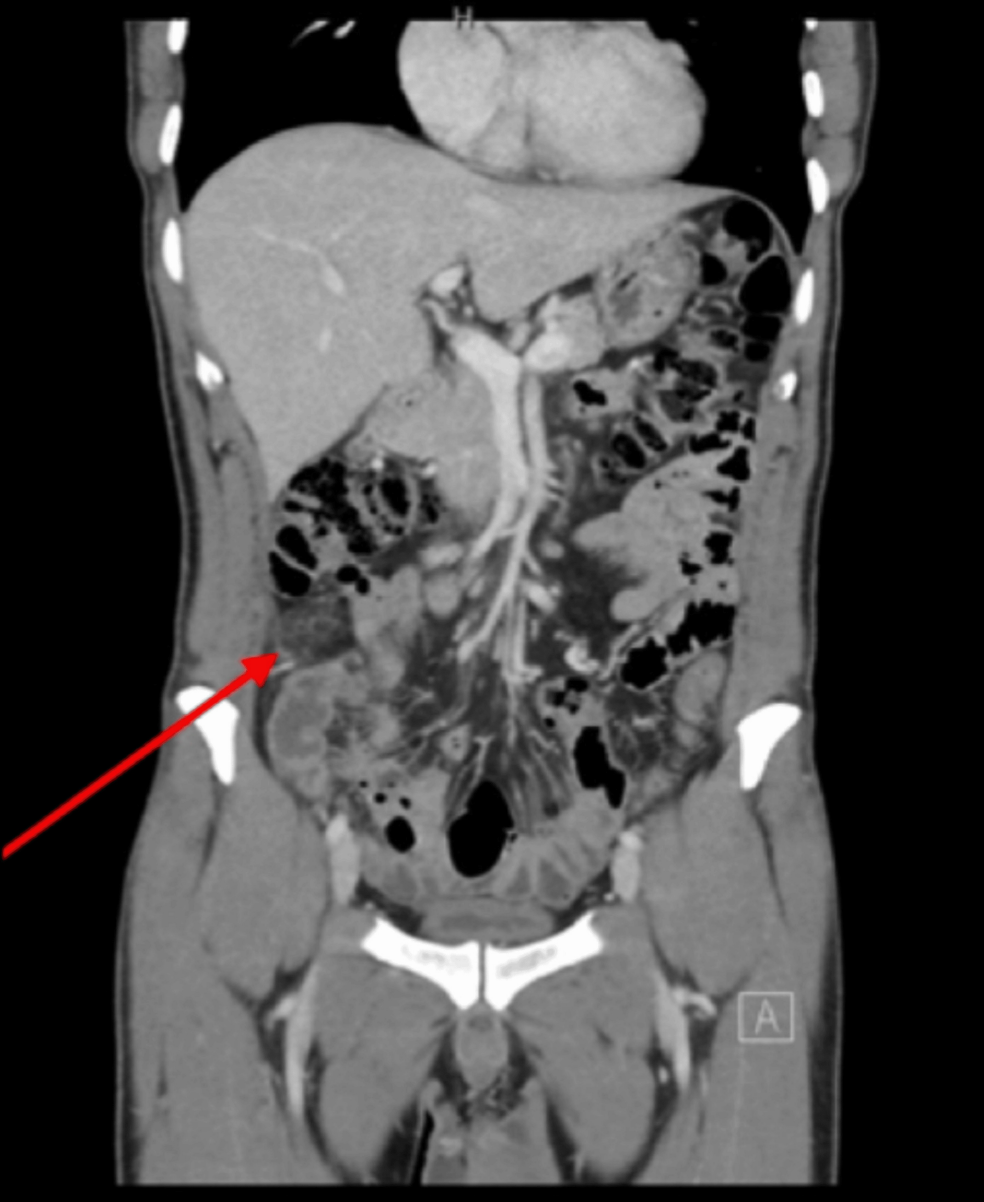
Contrast-enhanced CT of the abdomen and pelvis demonstrating primary OI Coronal contrast-enhanced CT image showing a focal area of heterogeneous fat stranding and inflammatory changes in the right lower quadrant within the greater omentum (red arrow), with no evidence of appendiceal inflammation or other intra-abdominal pathology. OI, omental infarction

**Figure 2:**
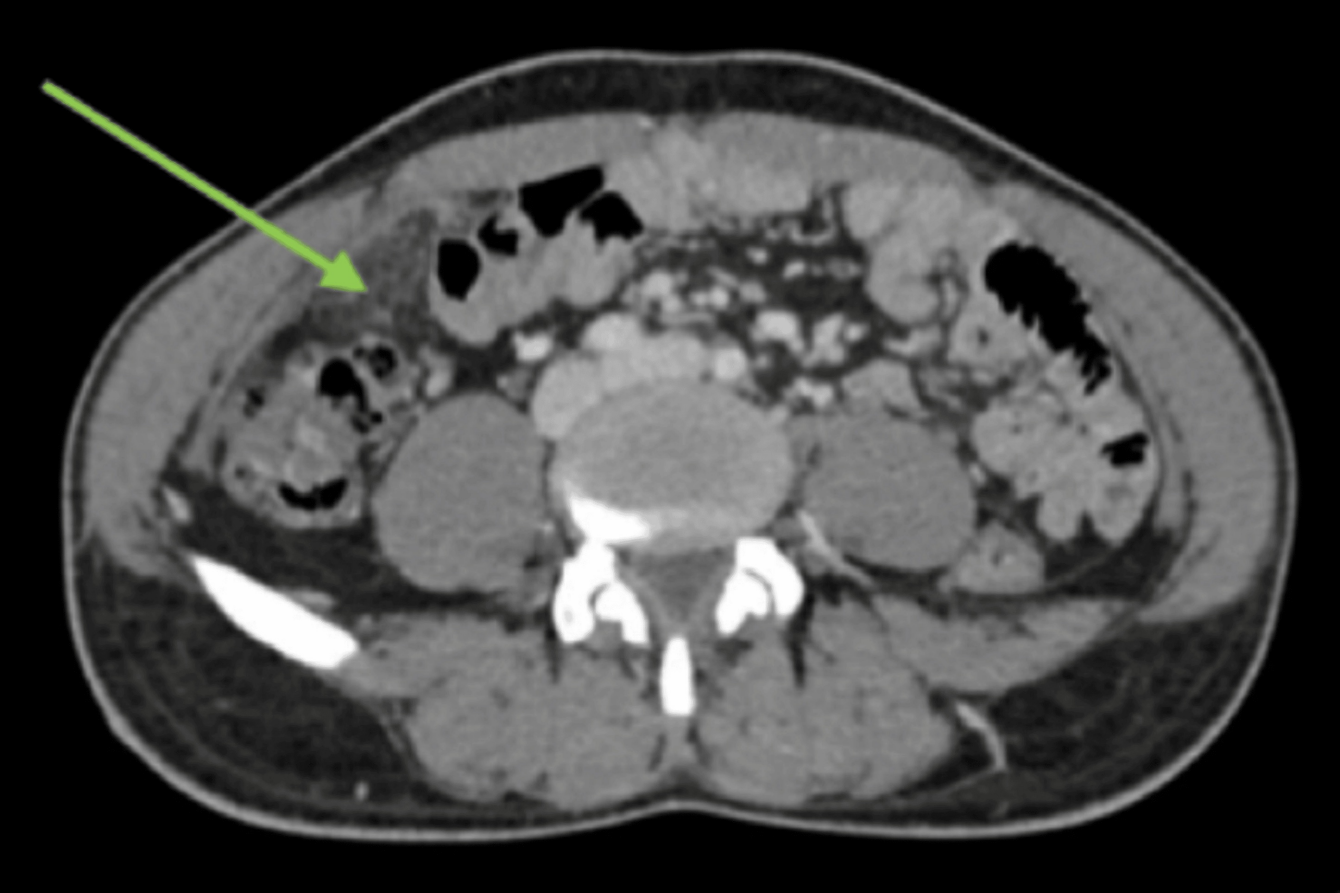
Axial contrast-enhanced CT of the abdomen demonstrating primary OI Axial contrast-enhanced CT image showing focal fat stranding and inflammatory changes in the right lower quadrant within the greater omentum (green arrow), consistent with OI. OI, omental infarction

Contrast-enhanced CT of the abdomen and pelvis demonstrated focal fat stranding and inflammatory changes within the omentum in the right lower quadrant, with no evidence of appendicitis or other intra-abdominal pathology (Figure 1 and Figure 2). A diagnosis of primary OI was made.

The patient was managed conservatively with analgesics and anti-inflammatory medications. He was discharged in stable condition with outpatient follow-up, and his symptoms resolved completely within one week.

## Discussion

OI is a rare and often underrecognized cause of acute abdominal pain [1]. This underrecognition is primarily due to its nonspecific clinical presentation and its close resemblance to more common surgical conditions such as acute appendicitis. Although CT can reliably establish the diagnosis, lack of initial clinical suspicion and historical reliance on intraoperative findings before widespread CT use have contributed to it being overlooked in clinical practice. It predominantly affects males and is most commonly observed in middle-aged adults [2]. The greater omentum is a large peritoneal fold that protects abdominal organs and limits infection. Vascular compromise of this fatty apron results in ischemia and necrosis of the affected segment [3].

OI is classified as primary or secondary. Primary OI occurs without preexisting pathology and is believed to result from spontaneous torsion, venous stasis, or thrombosis [4]. Predisposing factors include obesity, sudden body movements, and increased intra-abdominal pressure after heavy meals, which may promote torsion of the mobile omental segment. Secondary OI can result from adhesions, hernias, inflammatory processes, or prior surgeries [5].

The clinical features of OI are nonspecific and often mimic appendicitis or cholecystitis [2]. Pain is typically sudden in onset, localized to the right lower or upper quadrant, and usually unaccompanied by systemic signs such as fever or vomiting [3]. Normal laboratory results and the absence of peritoneal signs may help differentiate OI from more urgent surgical conditions.

Imaging, particularly CT, is pivotal for diagnosis [4]. Ultrasonography may reveal a hyperechoic, noncompressible mass, but CT remains the gold standard. Typical CT findings include a well-circumscribed fatty lesion with heterogeneous attenuation and surrounding inflammatory stranding confined to the omentum [5]. The “whirl sign,” representing twisted omental vessels, may be seen in cases of torsion. Differentiation from epiploic appendagitis is essential, as epiploic appendagitis is typically smaller, pericolonic, and adjacent to the colon rather than within the omental fat [2].

Most patients are treated conservatively with analgesics and nonsteroidal anti-inflammatory drugs [4]. Symptoms usually resolve within one to three weeks. Surgery is reserved for cases with diagnostic uncertainty, persistent symptoms, or complications such as abscess formation [5]. Laparoscopic resection, when performed, provides both definitive diagnosis and treatment.

Historically, OI was often discovered incidentally during laparotomy for suspected appendicitis [1]. However, with increased utilization of CT imaging, accurate preoperative diagnosis has become more common, allowing nonoperative management in many cases [4,5]. Early imaging has been associated with reduced hospital stay and avoidance of unnecessary appendectomy [4].

Several case reports in the literature have described primary OI presenting with clinical features mimicking acute appendicitis, particularly right lower quadrant pain with minimal systemic symptoms. Similar presentations have been documented in a series of patients in whom the diagnosis was established using CT imaging, highlighting its critical role in avoiding unnecessary surgical intervention [2]. Likewise, characteristic CT findings, including focal fat stranding within the omentum, have been described and aid in differentiating OI from other causes of acute abdomen [3,5].

In comparison, our case demonstrated a similar clinical presentation with localized right lower quadrant pain and absence of significant systemic symptoms. Although inflammatory markers were elevated (CRP 25.7 mg/L), imaging findings were consistent with previously reported cases, confirming OI with a normal appendix. This allowed for conservative management with complete symptom resolution, further supporting existing evidence that early CT imaging is essential in establishing the diagnosis and preventing unnecessary surgical intervention.

From an emergency medicine perspective, OI should be suspected in patients presenting with localized abdominal pain, minimal systemic symptoms, and normal laboratory investigations. Early CT imaging plays a crucial role in diagnosis and allows safe conservative management, preventing unnecessary surgical exploration.

## Conclusions

Primary OI is a rare but important differential diagnosis in patients presenting with localized abdominal pain, particularly when the clinical picture mimics acute appendicitis. Awareness of its characteristic radiologic appearance can facilitate timely diagnosis and support conservative management in selected patients. Early recognition of this entity may help avoid unnecessary surgical intervention, shorten hospital stay, and improve overall patient care in the emergency setting. In addition, increased awareness among emergency physicians and radiologists is essential to ensure appropriate diagnosis and management. Prompt use of imaging, particularly CT, plays a key role in confirming the diagnosis and guiding safe, nonoperative treatment.
